# Functions of Viroporins in the Viral Life Cycle and Their Regulation of Host Cell Responses

**DOI:** 10.3389/fimmu.2022.890549

**Published:** 2022-06-02

**Authors:** Xiaoyan Xia, Anchun Cheng, Mingshu Wang, Xumin Ou, Di Sun, Sai Mao, Juan Huang, Qiao Yang, Ying Wu, Shun Chen, Shaqiu Zhang, Dekang Zhu, Renyong Jia, Mafeng Liu, Xin-Xin Zhao, Qun Gao, Bin Tian

**Affiliations:** ^1^ Institute of Preventive Veterinary Medicine, Sichuan Agricultural University, Chengdu City, China; ^2^ Key Laboratory of Animal Disease and Human Health of Sichuan Province, Sichuan Agricultural University, Chengdu City, China; ^3^ Avian Disease Research Center, College of Veterinary Medicine, Sichuan Agricultural University, Chengdu City, China

**Keywords:** viroporins, function, viral life cycle, host cell response, interactions, inhibitors

## Abstract

Viroporins are virally encoded transmembrane proteins that are essential for viral pathogenicity and can participate in various stages of the viral life cycle, thereby promoting viral proliferation. Viroporins have multifaceted effects on host cell biological functions, including altering cell membrane permeability, triggering inflammasome formation, inducing apoptosis and autophagy, and evading immune responses, thereby ensuring that the virus completes its life cycle. Viroporins are also virulence factors, and their complete or partial deletion often reduces virion release and reduces viral pathogenicity, highlighting the important role of these proteins in the viral life cycle. Thus, viroporins represent a common drug-protein target for inhibiting drugs and the development of antiviral therapies. This article reviews current studies on the functions of viroporins in the viral life cycle and their regulation of host cell responses, with the aim of improving the understanding of this growing family of viral proteins.

## 1 Introduction

Viroporins are a class of small-molecule hydrophobic transmembrane proteins encoded by viruses, generally with 50-120 amino acid residues. A typical feature of viroporins is the presence of at least one transmembrane helix that anchors the protein into the membrane. Upon insertion into the membrane, their oligomerization produces hydrophilic channels or pores (1). Viroporins also have several characteristic structural motifs, including a set of basic residues (Lys or Arg) and an amphipathic α-helix, these basic amino acids are adjacent to the transmembrane domain and contribute to membrane binding, which controls the rhythm of viral reproduction for optimal spread by inducing membrane perforation at the correct cellular locations at different stages of the viral life cycle (2). Viroporins are essential for viral pathogenicity and replication and are involved in multiple processes including entry, uncoating, replication, assembly, and release in the viral life cycle (**Table 1** and **Figure 2**). In addition, the ion channel activity of viroporins can cause the homeostasis of intracellular ions (e.g., Na^+^, K^+^, Ca^2+^, Cl^-^) (91). The functional activities of viroporins will affect the host cells and participate in defensive signaling pathways after virus infection of host cells, including autophagy (**Figure 4**), apoptosis (**Figure 5**), cellular immune responses (**Figure 6**), ensuring the completion of virus replication by disrupting the host cell physiology (**Table 2**). Viroporins were first identified in several RNA viruses, such as protein 2B (P2B) in picornaviruses (203) and matrix protein 2 (M2) in influenza A virus (IAV) (204), and subsequently more and more viroporins have been studied and reported. In addition to the well-known viroporins such as poliovirus 2B, alphavirus 6K, HIV Vpu, hepatitis C virus (HCV) p7 [reviewed in (1, 205)], human astrovirus (HAstV) XP (89), dengue virus (DENV) NS2A and NS2B (61), ebola virus (EBOV) delta peptide (88), Norwalk Viruses (NV) NS1-2 (68), classical swine fever virus (CSFV) p7 (56), and bluetongue virus (BTV) NS3 (76) have been reported to have viroporin-like activity and are proposed to be members of the viroporin family.

**Table 1:** Viroporins and their roles in the viral life cycle.

Family	Virus	Viroporin	Amino Acid	Function in Viral Life Cycle	Ion Permeability	TMDs and Transmembrane Mode	Location	References
*Picornaviridae*	FMDV	2B	154	–	Ca^2+^	2, IIB	ER	(3, 4)
	PV	2B	97	Viral replication Viral release	–	2, IIB	Golgi, ER, Mitochondrion	(5, 6)
		3A	87	Viral replication	–	1, IB	ER	(7, 8)
	CVB	2B	99	Viral replication Viral release	Ca^2+^, H^+^	2, -	ER, Golgi, Mitochondrion	(9–11)
	EMCV	2B	151	–	Ca^2+^	2, -	Golgi	(12)
	EV71	2B	99	Viral replication Viral release	Cl^-^	2, -	Golgi, Mitochondrion	(13, 14)
	HRV	2B	97	–	Ca^2+^	–	ER, Golgi	(12, 15)
	DHAV-1	2B	119	–	Ca^2+^	1, IA	–	(16)
	HAV	2B	251	Viral replication	–	2, IIB	ER	(12, 17, 18)
*Coronaviridae*	MHV	E	83	Viral assembly Viral release	Na^+^, K^+^	1, IA	ERGIC, Golgi	(19, 20)
	SARS-CoV	E	76	Viral Assembly Viral release	H^+^, Na^+^ K^+^, Cl^-^, Ca^2+^	1, IA	ER, ERGIC, Golgi	(21–23)
		3a	274	Viral assembly Viral release	K^+^, Na^+^	3, -	Golgi, PM	(24, 25)
		8a	39	–	K^+^	1, -	Mitochondrion	(26, 27)
	IBV	E	108	Viral assembly Viral release	Na^+^, K^+^ H^+^	1, IA	Golgi	(28–32)
	HCoV-OC43	ns12.9	109	Viral assembly	K^+^	1, IB	ERGIC	(33)
	HCoV-229E	4a	133	Viral assembly Viral release	K^+^	3, -	ERGIC	(34)
*Togaviridae*	SINV	6K	55	Viral assembly Viral release	Ca^2+^	1, -	ER	(5, 35)
	SFV	6K	60	Viral assembly Viral release	Na^+^, K^+^ Ca^2+^	1, -	ER	(36)
	RRV	6K	62	Viral release	Na^+^, K^+^ Ca^2+^	1, IA	ER	(36, 37)
*Orthomyxoviridae*	IAV	AM2	97	Viral entry Genome uncoating Viral release	H^+^,	1, IA	Golgi	(38–41)
		PB1-F2	87/90	–	Ca^2+^, Na^+^	–	Mitochondrion	(42, 43)
	IBV	BM2	109	Genome uncoating Viral release	H^+^, K^+^, Na^+^	1, IA	Golgi	(44–46)
		NB	100	Viral assembly	–	1, IA	ER-Golgi/Perinucler region	(47, 48)
	ICV	CM2	115	Viral assembly Genome uncoating	Cl^-^	1, IA	ER	(49, 50)
	IDV	DM2	152	–	Cl^-^	1, IA	–	(51)
*Flaviviridae*	HCV	p7	63	Viral assembly Viral release	H^+^, Na^+^ K^+^	2, IIA	ER	(52–55)
	CSFV	p7	67	Viral release	Ca^2+^	2, IIA	ER	(56, 57)
	DENV	NS2A	218	Viral replication Viral assembly Viral release	–	–	ER, Mitochondrion	(58–60)
		NS2B	127	–	–	3, -	ER, Mitochondrion	(58, 61)
*Retroviridae*	HIV-1	Vpu	81	Viral assembly Viral release	K^+^, Na^+^	1, IA	TGN, PM, ER	(62–64)
*Paramyxoviridae*	RSV	SH	64/65	–	K^+^, Na^+^	1, IB	ER, Golgi	(65, 66)
	HMPV	SH	179	Viral entry	–	–	PM	(67)
*Caliciviridae*	TV	NS1-2	233	–	Ca^2+^	2, IIB	ER	(68)
	NV	NS1-2	341	–	–		ER	(68, 69)
*Phycodnaviridae*	PBCV-1	Kcv	94	–	K^+^	2, -	ER	(70, 71)
*Reoviridae*	RV	NSP4	175	Viral assembly Viral replication	Ca^2+^	3, -	ER	(72, 73)
	ARV	p10	98	Viral release	–	1, IA	Cell surface	(74, 75)
	BTV	NS3	229	Viral assembly Viral release	–	2, IIB	Golgi, PM	(76)
*Rhabdoviridae*	BEFV	α1	88	–	–	1, IA	Golgi	(77)
*Polyomaviridae*	SV40	VP2	352	Viral entry Viral assembly	–	–	Nucleoplasm	(78–80)
		VP3	234	Viral entry Viral assembly	–	–	Nucleoplasm	(78)
		VP4	125	Viral release	–	1, -	Cell nucleus	(79, 81, 82)
	JCV	agnoprotein	71	Viral replication Viral release	Ca^2+^	1, IB	ER, PM	(83)
	HPV	E5	83	Viral replication	–	3, -	ER, Golgi	(84–86)
	EBOV	Delta Peptide	40	Viral release	Cl^-^	–	–	(87, 88)
*Astroviridae*	HAstV	XP	112	Viral assembly Viral release	–	1, IA	TGNPM	(89)
*Herpesviridae*	HCMV	US21	243	–	–	7, -	ER	(90)

“-” represents “unidentified.” DHAV-1, duck hepatitis A virus; SFV, semliki forest virus; RRV, ross river virus; BEFV, bovine ephemeral fever virus; HCMV, human cytomegalovirus.

**Table 2:** Regulation of host cell responses by viroporins.

Virus	Viroporin	Regulation of host cell responses by Viroporins	Mechanism of host cell response regulation by viroporins	Viroporin action area	Inhibitor	References
FMDV	2B	Inhibiting protein secretion, disrupting intracellular Ca^2+^ homeostasis	–	–	Amantadine	(3, 92)
		Activating the NLRP3 inflammasome	Ion outflow	140-145 aa of the transmembrane region		(93)
		Inducing autophagy	changes in the Ca^2+^ content	–		(3)
		Antagonizing the host immune response	Interacting with CypA	115-118 aa		(94)
			Inhibiting the expression of RIP2 protein	N-terminal 105-114 and135-144 aa		(95)
			Inhibiting RIG-I and MDA5 protein expression	N-terminal 105 -114 and 135 -144 aa		(96, 97)
			Inhibiting phosphorylation of TBK1 and IRF3	–		(96)
			Inhibiting LGP2 expression	C-terminal 101-154 aa		(98)
			Inhibiting NOD2 expression	N-terminal 105-114 and 135-144aa		(99)
PV	2B	Inhibition of protein transport and disruption of intracellular Ca^2+^ homeostasis	Decrease in organelle Ca^2+^ concentration and increase in extracellular Ca^2+^ influx	–	enviroxime	(6, 12, 100, 101)
		Induction of apoptosis	–	–		(102)
	3A	Inhibition of protein transport	–	–		(103, 104)
		Antagonizing the host immune response	Impairing MHC class 1 antigen presentation	–		(105)
		Inducing autophagy	Inducing co-localization of LC3 and LAMP1	–		(106)
CVB	2B	Inhibition of protein transport and disruption of intracellular Ca^2+^	Decrease in organelle Ca^2+^ concentration and increase in extracellular Ca^2+^ influx	Cationic amphiphilic α helix	–	(107)
		Inducing autophagy	–	36aa-83aa region, valine 56 is important		(108)
		Inhibition of apoptosis	Manipulation of intracellular Ca^2+^ homeostasis	–		(9)
EMCV	2B	Disruption of intracellular Ca^2+^ homeostasis	Reducing Ca^2+^ concentration in the endoplasmic reticulum	–		(12)
		Activating the NLRP3 inflammasome	Disturbing intracellular Ca^2+^ concentration	–		(109)
		Stimulating immune response	Triggering mtDNA translocation to the cytoplasm	–		(110)
EV71	2B	Inducing apoptosis	Recruiting Bax, promoting its redistribution	N-terminal 23- 35 aa	DIDS	(13, 14, 111)
		Antagonizing the host immune response	Induction of KPNA1 degradation	N-Terminal Domain		(112)
			Inhibiting ILF2 expression, promoting ILF2 translocation	–		(113)
HRV	2B	Inhibition of protein transport	–	–	–	(12)
		Activation of NLRP3 and NLRC5 inflammasomes	Activating PERK and ATF6	–		(114)
		Induction of apoptosis		–		(15)
DHAV-1	2B	Disruption of intracellular Ca^2+^ homeostasis	–	–		(16)
		Inducing incomplete autophagy	–	–		(16)
HAV	2B	Disruption of intracellular Ca^2+^ homeostasis	–	–		(12)
		Antagonizing the host immune response	Interference with IRF-3 phosphorylation	–		(115)
MHV-A59	E	Inducing apoptosis	–	–		(116)
SARS-CoV	E	Affecting protein transport	–	YXXΦ motif	Gliclazide, Memantine, Amantadine, HMA, Tretinoin, Rutin, doxycycline	(117–121)
		Activating the NLRP3 inflammasome	Disturbing intracellular Ca^2+^ concentration	Disturbing intracellular Ca^2+^ concentration		(122)
		Triggering an inflammatory response	Interacting with syntenin to activate p38 MAPK	C-terminal PDZ-binding motif		(123)
		Inducing apoptosis	Interacting with Bcl-xL	–		(124)
		Inhibition of apoptosis	Downgrading IRE-1	–		(125)
	3a	Activating the NLRP3 inflammasome	–	–	Kaempferol derivatives, Emodin	(126–128)
		Triggering an inflammatory response	Activation of JNK and NK-kappaB	–		(129, 130)
		Inducing autophagy	Triggering lysosomal damage and dysfunction,	–		(Yuan 131)
		Inhibiting autophagy	Blocking the assembly of SNARE complexes	Transmembrane region		(132)
		Inducing apoptosis	–	K^+^ channel activity		(133, 134)
			Activation of p38 MAP kinase	–		(135)
		Antagonizing the host immune response	Inhibition of IFNAR1 Trigger mtDNA translocation to the cytoplasm	–		(136)
	8a	Inducing apoptosis	Disturbance of Mitochondrion membrane potential	–	–	(27)
IBV	E	Inhibiting protein transport	–	Hydrophobic domain	–	(29)
		Inducing apoptosis	Activation of ER stress	–		(137)
IAV	AM2	Alteration of cell membrane permeability	–	–	Amantadine, Rimantadine, Tretinoin	(5, 138, 139)
		Activating the NLRP3 inflammasome	Disturbance of intracellular ion concentration	–		(140)
		Inhibiting autophagy	Interacting with LC3 or Beclin-1; blocking fusion of autophagosomes and lysosomes	M2 Transmembrane region; LC3 interacting region (LIR); N-terminal 60 aa		(141–144)
		Inducing autophagy	Triggering extracellular Ca^2+^ influx-dependent ROS production	–		(145, 146)
			Decreasing AKT phosphorylation	–		(147)
		Inducing apoptosis	Blocking autophagosome maturation	–		(148)
			Forming stable complexes with Hsp40 and P58(IPK) to enhance PKR autophosphorylation	–		(149)
		Stimulating immune response	Triggering mtDNA translocation to the cytoplasm	–		(110)
			Interacting with MAVS	His37		(145)
	PB1-F2	Regulation of RLRP3 inflammasome activation	–	C-terminal 40 aa (located to 62nd, 75th, 79th, and 82nd aa)	–	(150–155)
		Inducing apoptosis	Interacting with ANT3 and VDAC1 to reduce Mitochondrion membrane potential	Interaction of C-terminus with ANT3, N-terminus and C-terminus with VDAC1		(152, 155, 156)
		Exacerbating innate immune response	Induction of IFN-β, leading to cytokine dysregulation	62-70 aa (LSLRNPILV)		(157, 158)
		Antagonizing the host immune response	Combine with MAVS and reduce MMP	C-terminal		(159)
			Interference with the RIG-I/MAVS complex	–		(160)
			Blocking K63-polyubiquitination and MAVS aggregation and promoting MAVS degradation	–		(161)
			Inhibition of MAVS protein expression	C-terminal 38-87 aa		(162)
			Degradation of MAVS	C-terminal LIR motif		(163)
			Decrease Δψm	–		(155)
IBV	BM2	Inhibition of apoptosis	Inhibiting p53 activity	Cytoplasmic domain		(164)
		Inducing apoptosis	Forming stable complexes with Hsp40 and P58(IPK) to enhance PKR autophosphorylation			(149)
HCV	P7	Inhibition of pro-inflammatory response	Activating STAT3 and ERK	–	Amantadine, Rimantadine, HMA, BIT225	(165–167)
		Activating the NLRP3 inflammasome	–	–		(168)
CSFV	p7	Disruption of intracellular Ca^2+^ homeostasis	–	–	Amantadine, Verapamil	(56, 57)
DENV	NS2A	Activating the NLRP3 inflammasome	Disturbing intracellular Ca^2+^ concentration	–	–	(58)
		Antagonizing the host immune response	Blocking STAT1 phosphorylation	–		(169)
			Blocking TBK1/IRF3 phosphorylation	–		(170)
			Cutting STING	–		(171)
	NS2B	Activating the NLRP3 inflammasome	Disturbing intracellular Ca^2+^ concentration	–		(58)
		Antagonizing the host immune response	Degradation of cGAS	–		(172)
			Cutting STING	–		(173)
HIV-1	Vpu	Inducing apoptosis	Inhibition of p53 ubiquitination	β-TrcP binding motif	BIT225	(174–176)
		Antagonizing the host immune response	Downregulating BST-2	Conserved serine in the cytoplasmic domain		(63, 64)
			Downregulation of CD4 and BST-2	Cytoplasmic domain		(177)
			Degradation of CD47	Transmembrane region		(62)
			Inhibiting MAVS expression	–		(178)
			Inhibiting STAT1 phosphorylation	–		(179)
			Inhibition of NF-κB transcription	Arginine residues in the cytoplasmic domain		(180)
RSV	SH	Activating the NLRP3 inflammasome	Disturbance of intracellular ion concentration	–	pyrnin B	(66, 181)
		Inhibition of apoptosis	–	–		(182)
		Antagonizing the host immune response	Inhibiting p65 phosphorylation	–		(183)
HMPV	SH	Antagonizing the host immune response	Inhibition of NF-κB transcription	–	–	(184)
		Antagonizing the host immune response	Inhibition of STAT1 expression and phosphorylation	–		(185)
TV	NS1-2	Disruption of intracellular Ca^2+^ homeostasis	–	–	**-**	(68)
NV	NS1-2	Antagonizing the host immune response	Decreasing TLR-4, -7, -8 and -9 expression	–	–	(186)
			Interaction with VAP-A	NS1 structure domain		(187, 188)
RV	NSP4	Disruption of intracellular Ca^2+^ homeostasis	–	–		(189, 190)
		Inducing autophagy	Activating CaMKK-β signaling pathway; targeting IGF1R; blocking PI3K/Akt pathway	–		(191–193)
BTV	NS3	Antagonizing the host immune response	Interacting with BRAF to enhance the MAPK/ERK pathway	–	–	(194)
			Targeting STAT1	–		(195)
			Targeting STAT2	PPRY structure domain		(196)
JCV	agnoprotein	Promoting apoptosis	–	–	–	(197)
HPV	E5	Inhibit endosomal acidification	–	–	Rimantadine	(86, 198, 199)
		Inhibition of apoptosis	Decreasing Bax protein expression	–		(200)
		Antagonizing the host immune response	Down-regulation of surface MHC class I activity	TMD1(LL1-LL4) motif		(201)
			Inhibiting IFN-κ transcription	–	–	(202)

“-” represents “unidentified.”

**Figure 1:**
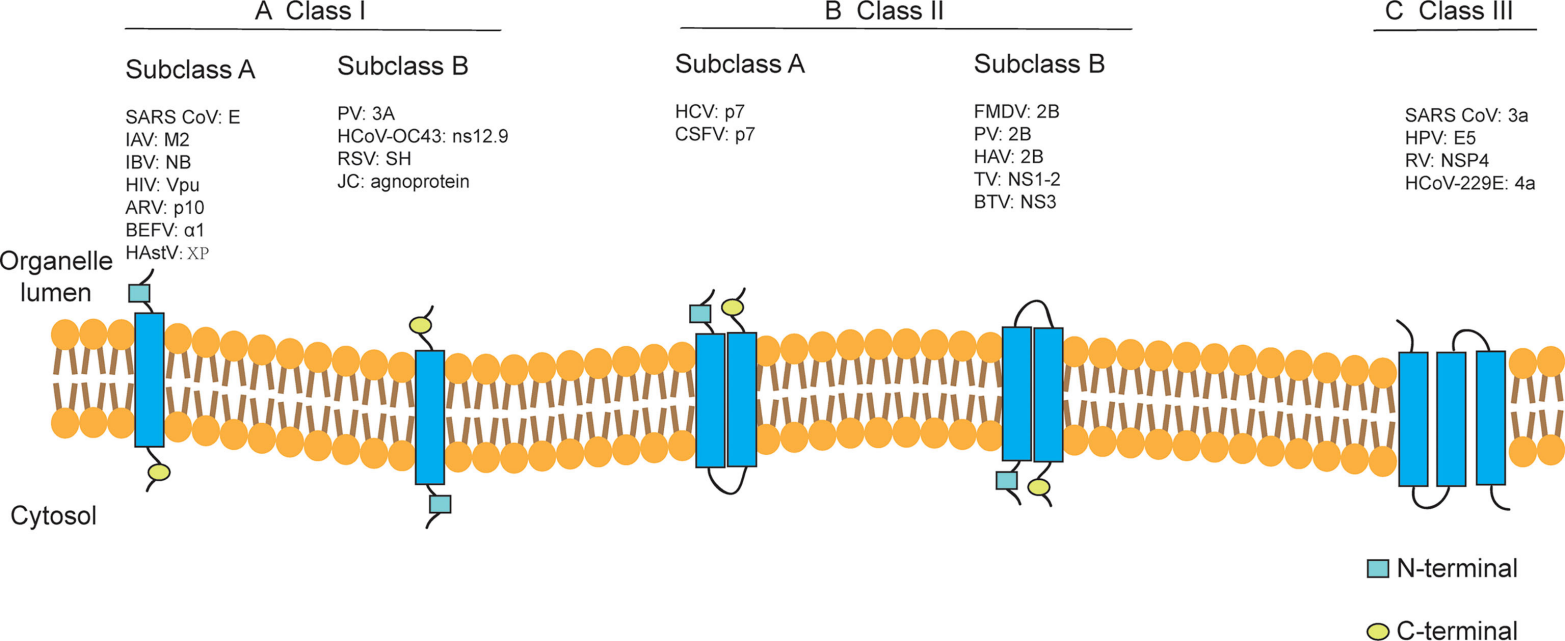
Classification of viroporins according to the number of transmembrane domains and the membrane topology of the constituent monomers. Class I and Class II viroporins have one and two TMD, respectively. **(A)** Class I A viroporins have their N-termini facing the lumenal side while Class I B have their N-termini in the cytosolic side. **(B)** Class II A viroporins have both the N- and C-termini in the lumenal side while Class II B have them facing the cytosol. **(C)** Class III viroporin with three TMDs. HCoV-OC43, human coronavirus OC43; TV, tulane virus. Figure adapted from (205).

Depending on the internal nucleic acids of the viruses containing viroporins, they can be classified into viroporins encoded by DNA viruses (e.g., JC virus agnoprotein (83), human papillomavirus E5 (86), simian virus 40 VP4 (82) and viroporins encoded by RNA viruses (e.g., PV 2B (203, 206), IAV M2 (204), HCV p7 (54), BTV NS3 (76). According to the number of hydrophobic transmembrane domains (TMD) of viroporins, they are divided into two major classes: Class I and Class II, which can be further divided into subclasses A and B according to their different transmembrane modes (205) (**Figure 1**). Viroporins containing three hydrophobic transmembrane regions have also been identified in recent years, such as rotavirus (RV) NSP4 (189), the human papillomavirus(HPV) E5 protein (85), human coronavirus 229E (HCoV-229E) 4a (R. 34) and SARS-CoV 3a (24), but no further classification of such proteins has been performed.

Viroporins can form selective ion channels in the host cell membrane that mediate the transport of physiologically relevant ions (e.g., Na^+^, K^+^, Ca^2+^, Cl^-^ or H^+^) (**Table 1**). For example, the IAV M2 protein can form a proton channel (41), while the Kcv protein encoded by paramecium bursaria chlorella virus 1(PBCV-1) is a K^+^ selective channel (71). However, most viroporins generally exhibit weak ion selectivity, and these channels generally do not show a preference for specific ions. For example, IAV PB1-F2 viroporin can generate conductance in the lipid bilayer without apparent selectivity and conducts both Ca^2+^ and Cl^-^ (43). The SARS-CoV E protein is more selective for monovalent cations (Na^+^ and K^+^) than monovalent anions (Cl^-^) (207).

In addition, immunolocalization studies on virus-infected cells showed that most of the viroporins were localized on various intracellular organelles (e.g., Golgi, endoplasmic reticulum), while fewer are detected on the plasma membrane (**Table 1**). The names, amino acid sizes, roles in the viral cycle, and classification of the members of the viroporin family that have been proposed as a result of the studies are reported in **Table 1**. This article discusses the functions of currently reported viroporins in the viral life cycle and their regulation of host cell responses, emphasizing their potential as antiviral targets.

## 2 The Role of Viroporins in the Viral Life Cycle

**Figure 2:**
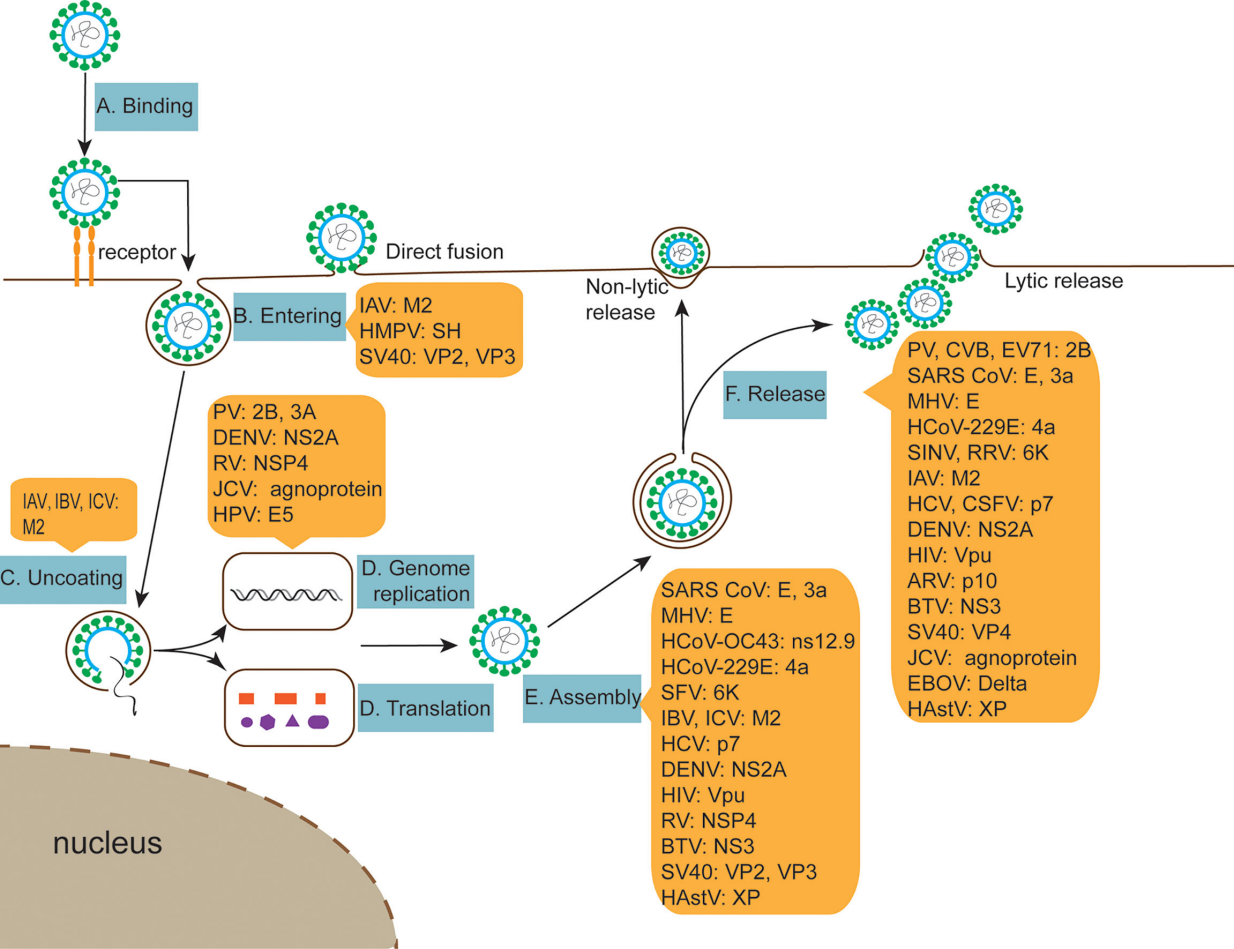
The role of viroporins in the viral life cycle. **(A, B)** Viroporins facilitate viral penetration of host plasma membrane into cells. **(C)** Viroporins trigger conformational changes in the virus, releasing the genome. **(D)** Viroporins-mediated viral replication. **(E)** Viroporins facilitate the assembly of new viral nucleic acids with protein capsids. **(F)** Viroporins promote virus release from host cells by budding or lysis.

Since viruses are obligate intracellular pathogens, they must depend on host cells for reproduction and metabolism. The life cycle of viruses varies greatly depending on the type and class of virus, but they follow the same basic stages of viral replication, i.e., adsorption, entry, uncoating, replication, assembly, and release. Although viroporins are involved in different stages of the viral life cycle (**Table 1** and **Figure 2**), most viroporins are mainly involved in the later steps of the viral life cycle, such as assembly and release, as far as the current study has found. Viruses cannot complete proper assembly and release when function of viroporins is disrupted.

### 2.1 Viral Entry and Uncoating

For infection to occur, the virus must first bind to and penetrate the host plasma membrane to deliver genetic material to the cytoplasm for replication. Enveloped and non-enveloped viruses employ different strategies to achieve the same goal. Viroporins, which are a structural component in enveloped viruses, actively facilitate the viral entry process, such as the influenza virus M2 protein. The influenza virus binds to the cell surface and is internalized into endosomes, and its encoded viroporin M2 is located in the viral lipid bilayer and is critical for infection. M2 acts as a proton-conducting channel in the viral envelope, supporting acidification of the viral interior in the endosome (208, 209). This process drives a series of conformational changes in the viral hemagglutinin (HA) protein, leading to the fusion of the viral envelope with the endosomal membrane and the delivery of nucleoprotein complexes into the cytoplasm (210). Acidification of M2 is also thought to promote the structural rearrangement of viral particles, which is required for the efficient uncoating of viral RNAs in the cytoplasm (45). Relevant studies have shown that the HMPV SH protein also has viroporin activity, which can regulate the fusion protein function during virus infection and may play a role in HMPV entering cells (67).

Non-envelope virions lack viroporins as structural components, and the involvement of such proteins in the process of virus entry into cells has been less well reported. A study found that antiretroviral (ARV) p10 is shown to play a key role in its viral fusion process, and the expression of p10 induces extensive cell-cell fusion in transfected cells. Thus, p10 is not only the first non-enveloped viral protein capable of promoting fusion from within, but also the first non-structural viral protein capable of inducing cell-cell fusion (75). Non-enveloped simian virus 40 (SV40) encodes three proteins with viroporin activity, VP2, VP3, and VP4, which play an important role in the life cycle of SV40. During infection, SV40 binds to the cell surface and is endocytosed into caveolin-coated vesicles (211) and subsequently translocated to the ER, where capsid reorganization and entry of viral particles into the cytoplasm occurs. VP2 and VP3 can integrate into the ER membrane and play an important role in the uncoating stage of the viral genome (78, 79). In addition to triggering viral particle infection, several viroporins encoded by influenza virus and SV40 play other roles in the viral life cycle, as will be discussed later.

### 2.2 Viral Replication and Assembly

After the release of the viral genome, the virus controls cellular proteins and organelles to achieve replication. Studies have shown that rearranged membranes are utilized during viral replication (104), and picornaviruses induce rearrangement of the host cell inner membrane to create structures that serve as functional scaffolds for genome replication (212). One of the most striking morphological changes that can be observed in enterovirus-infected cells is the massive accumulation of small ER and Golgi membrane vesicles in the cytoplasm (213). These vesicles are the site where viral RNA replication occurs. The 2BC protein has been implicated in the accumulation of these vesicles, where mutations that interfere with the pore formation capacity of 2B lead to defects in the early stages of viral RNA replication (213). Suhy et al. found that the poliovirus (PV) 2B protein alters cell membrane permeability and together with the 3A protein induces significant rearrangement of the intracellular membrane (104). PV 2B and 3A viroporins can also inhibit the cellular protein secretion pathway by disassembling the Golgi complex or blocking ER-Golgi transport, leading to the accumulation of membrane vesicles in the cytoplasm (101, 214). In addition, polioviruses harboring mutations in the 3A protein result in a marked reduction in positive-strand RNA synthesis (8). Picornavirus 2B protein also facilitates viral release by increasing the permeability of the plasma membrane, which will be discussed in the next section.

Throughout the replication cycle of coronaviruses, viruses use significant rearrangements of the host membrane for replication, protein expression, assembly, and release. The coronavirus E protein may also promote membrane rearrangement. When expressed alone in BHK-21 cells, mouse hepatitis virus (MHV) E can drive the intracellular formation of ERGIC-derived electron-dense membranes (19). The higher oligomers of infectious bronchitis virus (IBV) E are required for the production of virus-like particles (VLPs), suggesting that this form of protein is involved in the assembly of viral particles (215). Studies have shown that the MHV E protein is palmitoylated after translation, which contributes to the assembly of virions (216). The M, E, and N structural proteins of SARS-CoV are required for efficient assembly, transport, and release of virus-like particles (23). Recombinant CoVs (rCoVs) lacking the E gene (ΔE) exhibit abnormal morphology (217), suggesting that the E protein is involved in the assembly process. The function of the E protein is not to coordinate viral assembly but to induce bending of the viral envelope membrane so that the CoV particles acquire their characteristic spherical shape and morphology. Another important role of the coronavirus E protein is to regulate the pH within the lumen of intracellular organelles (e.g., the Golgi apparatus). For example, expression of the E protein of infectious bronchitis virus increases the pH within the organelle, induces neutralization of the Golgi pH, which results in a protective effect, allows isolation of the IBV spike-in protein from protein hydrolysis, and promotes viral assembly and release (32). Furthermore, the colocalization of ORF 3a with the M and E proteins essential for viral assembly suggests that ORF3a is important in the assembly or budding of SARS-CoV (25).

The rotavirus (RV) NSP4 protein also plays an important role in the viral assembly process. NSP4 manipulates the autophagic membrane trafficking process to take viral protein-containing membranes and transport them to viral replication sites for infectious particle assembly (191). The increase in cytoplasmic Ca^2+^ concentration mediated by NSP4 viroporin activates specific Ca^2+^-inducible signaling pathways to initiate autophagy, which allows the transport of the NSP4 and VP7 proteins to the virion for subsequent viral assembly (192). NSP4 binds to immature particles in the virion and triggers particle budding through these membranes to facilitate the assembly of capsid proteins on the particles to form mature infectious viral particles. Lopez et al. showed that small interfering RNAs that inhibit NSP4 expression in rotavirus-infected cells affect the distribution of other viral proteins, mRNA synthesis, and virion formation for viral RNA replication, suggesting a previously unrecognized function for NSP4 in RV replication (72).

The M2 protein is required for influenza virus assembly and budding. When viral budding begins, M2 is located at the edge of the budozone and induces a negative membrane curvature (218), thereby stabilizing the HA and M-induced positive membrane curvature in the budozone. Experimental results suggest that targeting M2 away from the apical plasma membrane may disrupt influenza virus assembly, budding, and replication (219). Phosphorylation of influenza virus CM2 promotes efficient virus replication (50). Deletion of CM2 results in impaired packaging and uncoating of virus-like particles (VLPs) and recombinant influenza viruses (49). The cytoplasmic structural domain of the BM2 protein performs the function of M1 binding to the viral ribonucleoprotein complex alone at the viral particle budding site (44, 220) and plays an important role in viral assembly. IAV M2 ubiquitination plays an important role in the production of infectious viruses by coordinating the efficient packaging of the viral genome into viral particles and the timing of virus-induced cell death (221). In addition, HCV p7 protein and HIV Vpu protein have also been shown to be involved in their viral assembly and release processes (52, 55, 62–64). The positively charged residue R84 in the dengue virus NS2A protein is essential for both viral RNA synthesis and intracellular viral particle assembly and maturation (60). Lulla et al. explored the role of the human astrovirus X protein in the viral life cycle by knocking it out and showed that the X protein may play a role in viral particle formation and viral release (89).

### 2.3 Viral Release

After assembly into infectious viral particles, viruses are released from host cells by budding (e.g., IAV, coronaviruses) or lysis (e.g., picornavirus, SV40). Alterations in plasma membrane permeability are important for cell lysis and the release of viral progeny (203). Enteroviruses are non-enveloped viruses that require the lysis of host cells to release newly formed virions. The expression of Enterovirus 2B protein disrupts intracellular Ca^2+^ homeostasis by progressively enhancing membrane permeability, leading to increased plasma membrane permeability and ultimately to membrane damage and release of the virus (6). For other viruses that are non-enveloped or are enveloped only by a protein coat, viral release often involves cell membrane perforation. For example, SV40 VP4 facilitates virus release by forming pores of approximately 3 nm in diameter in the host cell membrane to penetrate the membrane (82).

For enveloped viruses, budding and division are typically used to release virus from infected cells, and the mechanisms involved in the release process vary from virus to virus. During influenza virus release, the M2 protein is located at the neck of the budding virion, and the amphiphilic helix inserts into the membrane, inducing bending of the positive membrane, and finally pinching off the budding virion (218, 222). Mutations or deletions in the M2 cytoplasmic tail structural domain (CTD) significantly impair the assembly of viral proteins and genomic fragments into viral particles and viral particle release (218, 223, 224). A recent study found that the reduced hydrophobicity of the 91-94 motif of the IAV M2 protein significantly affected the budding ability of the M2 protein and compromised the bilayer membrane integrity of mutant viruses. It was suggested that the hydrophobic residues of the intracellular domain of the M2 protein play an important role in the release and membrane integrity of influenza virus (225). Several studies have shown that the coronavirus E protein is important for viral particle release. IBV E proteins have been shown to function in the secretory pathway, altering the luminal environment and rearranging secretory organelles, ultimately facilitating the efficient transport of viral particles (29–32). The E protein targets the Golgi apparatus and directs the release of virus-like particles (28). Mutations introduced into the HD of MHV or IBV affect the release of infectious particles from the cell (20, 29, 31). These findings suggest that IBV and MHV E viroporins may mediate viral release through their ion channel activity. In addition, Lu et al. found a significant reduction in viral release in cells transfected with ORF3a-specific siRNA (24), suggesting that the 3a protein may act as an ion channel promoting the viral release, but the mechanism by which the 3a protein affects virus release requires further study.

Unlike other viruses that mature in the lumen of the ER, RV is not released *via* the classical cytosolic pathway. Instead, recent data suggest that this virus may be transported from the ER directly through vesicles to the apical plasma membrane of polarized epithelial cells without passing through the Golgi complex. RV NSP4 interacts with immature particles to trigger outgrowth and synthesis into ER transmembrane proteins, and this rotavirus “budding” maturation process occurs through autophagy-hijacked COPII vesicle membranes (226). BTV NS3 protein of the same family as rotaviruses may function as the membrane protein of enveloped viruses, responsible for intracellular trafficking and budding of viral particles. Therefore, the NS3 protein is considered to act as a bridge between mature virions and cellular proteins during viral shedding (227). Recent studies have found that filovirus delta peptides function as viroporin to enhance the release of viral particles across host cell membranes (88). In addition, other viroporins such as alphavirus 6K protein, HCV p7 protein, HIV Vpu protein also play an important role in the virus release process (36, 55, 228) (**Table 1** and **Figure 2**).

## 3 Regulation of Host Cell Responses by Viroporins

**Figure 3:**
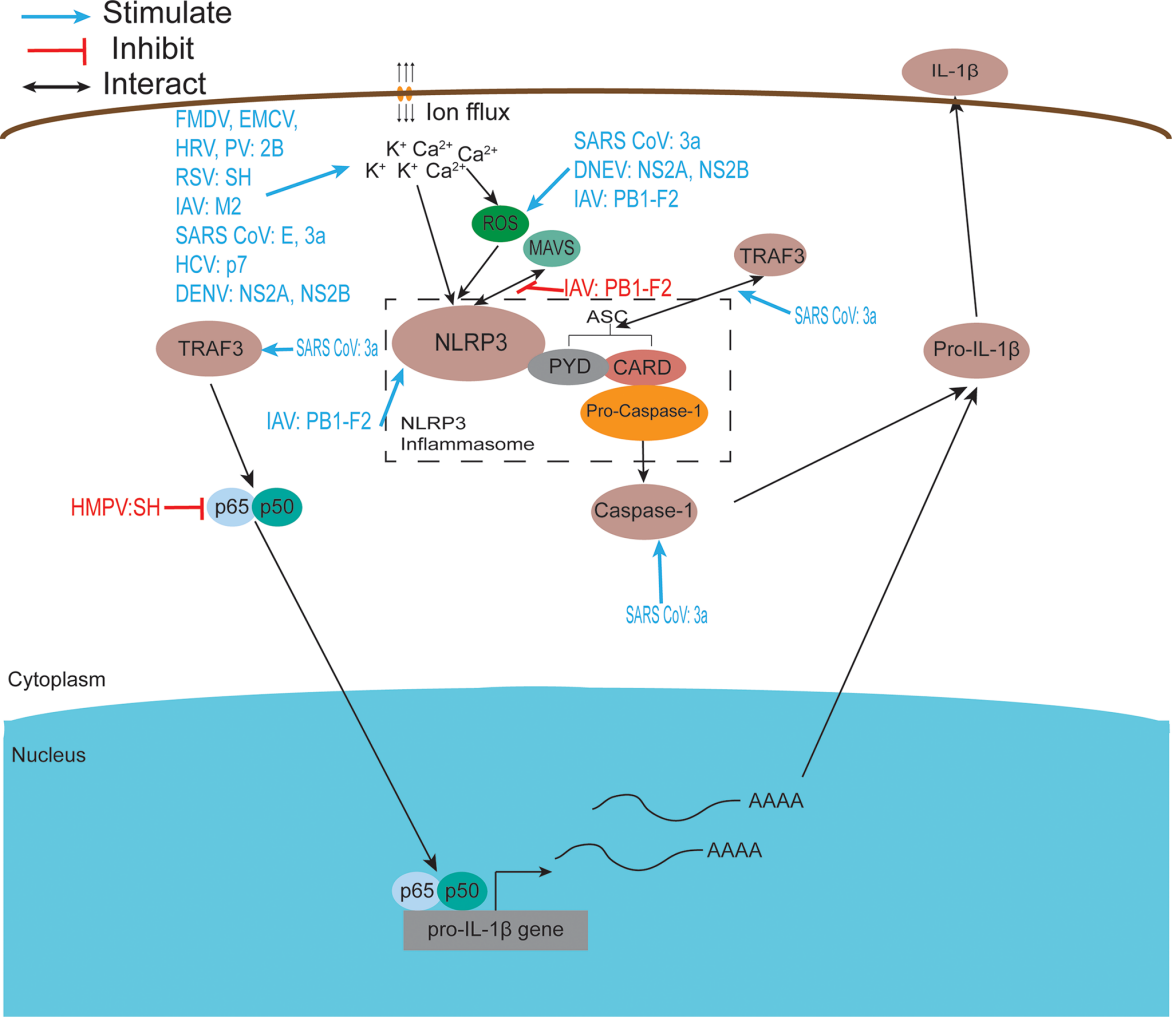
Viroporins regulate inflammasome activation. The NACHT, LRR, and PYD domain-containing protein 3 (NLRP3) inflammasome is an oligomeric complex composed of the NOD-like receptor NLRP3, the adaptor protein ASC, and Caspase-1 (229). Mitochondrial damage, protein aggregation, and abnormal ion concentrations caused by viral infection can activate the NLRP3 inflammasome leading to the secretion of IL-1β and IL-18. Most viroporins activate the NLRP3 inflammasome by disturbing intracellular ion concentrations. Some viroporins can activate NLRP3 through mitochondrial damage and increased ROS production.

**Figure 4:**
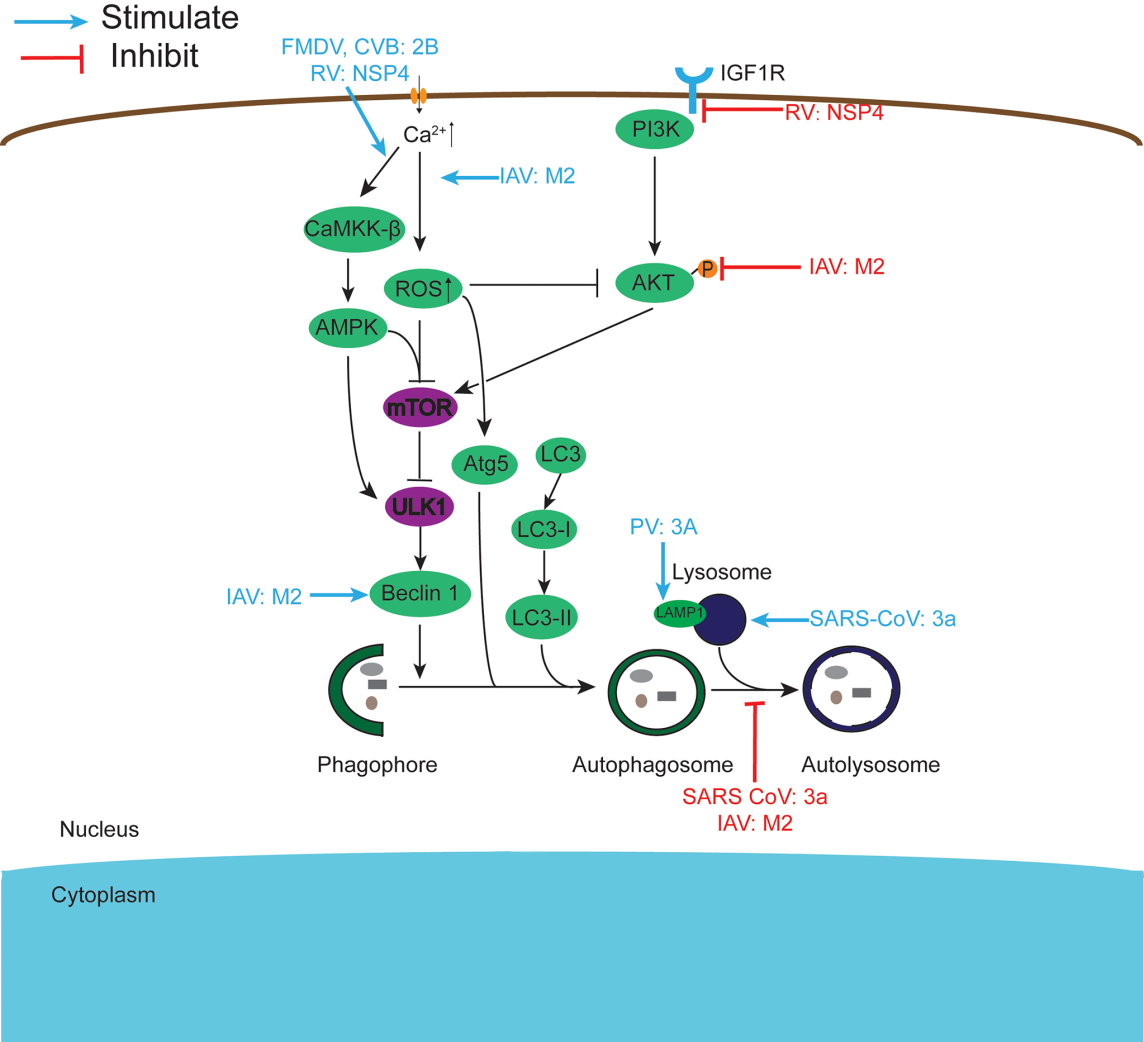
Viroporins regulate autophagy. Autophagy can be regulated by the PI3K-AKT-mTOR signaling pathway and the AMPK-TSC1/2-mTOR signaling pathway. Viroporins can regulate autophagy by regulating upstream signaling cascades, interfering with the formation of autophagosomes to activate, inhibit autophagy and fuse with lysosomes, and interact with key molecules of autophagy.

**Figure 5:**
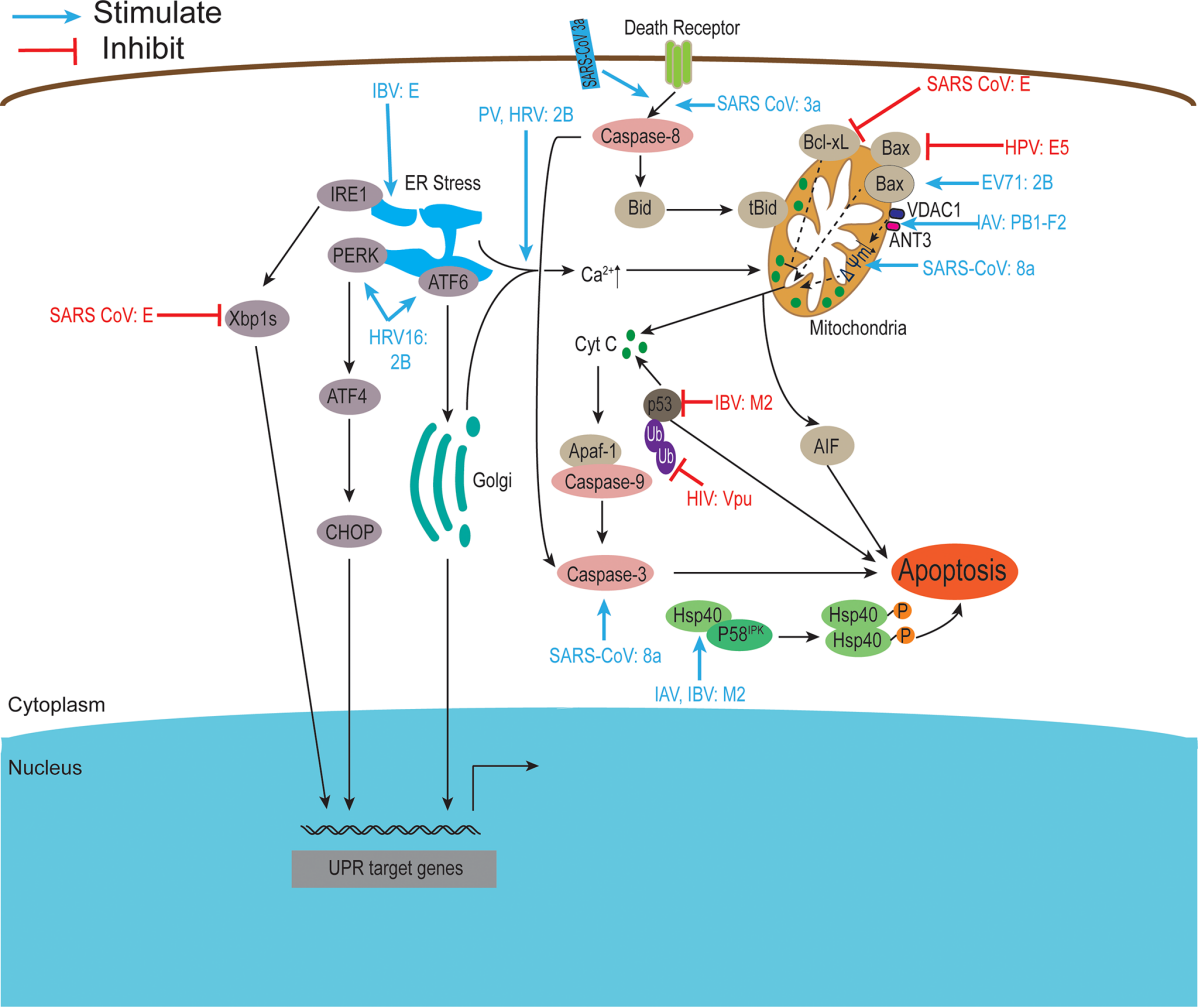
Viroporins regulate apoptosis. Apoptosis can be activated through two major signaling pathways, the death receptor-mediated pathway, and the mitochondrial pathway. Viroporins can induce apoptosis by changing calcium ion concentration, reducing mitochondrial membrane potential, recruiting apoptosis-related factors, and activating endoplasmic reticulum stress. Some viroporins can also inhibit apoptosis. Ub, Ubiquitin. Figure adapted from (230).

**Figure 6:**
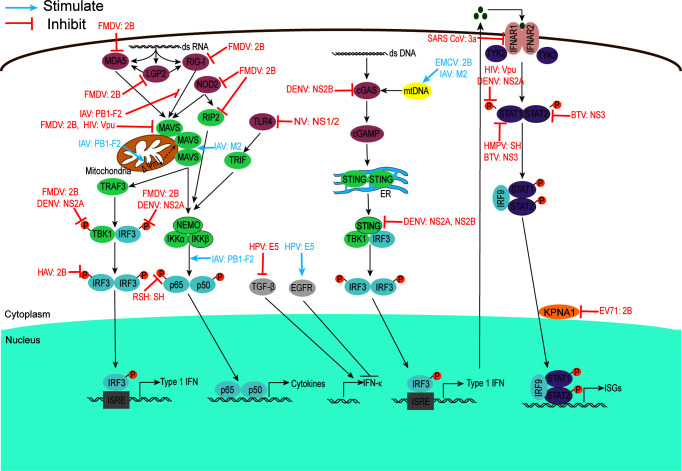
Viroporins regulate host immune responses. viroporins modulate host cell immune responses by interfering with PRRs recognition, interfering with bridging molecules, kinases, and downstream effectors in the innate immune signaling pathway, and interfering with IFN-mediated signaling. Figure adapted from (231).

Viroporins encoded by viruses can affect a variety of host cell responses (**Table 2**), such as altering cell membrane permeability, activation of the NLRP3 inflammasome (**Figure 3**), regulating apoptosis (**Figure 4**) and autophagy (**Figure 5**), and affecting host immune responses (**Figure 6**). Viroporins promote their replication and proliferation by regulating these responses.

### 3.1 Alteration of Host Cell Membrane Permeability and Disruption of Intracellular Ion Concentration Balance

Induction of changes in cell membrane permeability is a key function of viroporins (57, 83, 206, 207, 232). In addition to this, many virus-encoded viroporins can allow different ions (e.g., H^+^ and K^+^) across the cell membrane, affecting the concentration of ions inside and outside the cell as well as the membrane permeability to these ions. After infection of cells by picornavirus, increased membrane permeability leads to disruption of the extracellular ion gradient and thus disrupts the host intracellular ion concentration balance, in which 2B viroporin plays an irreplaceable role. 2B proteins such as foot-and-mouth disease virus (FMDV), PV, and Coxsackievirus B (CVB) can increase intracellular Ca2^+^ ion concentration, but by different mechanisms. The expression of CVB3 and PV 2B proteins leads to a decrease in Ca^2+^ concentration in the ER and Golgi complex as well as in Ca^2+^ uptake by mitochondria. At the same time, an increased influx of extracellular Ca^2+^ leads to increased cytoplasmic Ca^2+^ levels (9, 12). Similarly, the expression of human rhinovirus (HRV) 2B protein showed a decrease in Ca^2+^ concentration in the ER and Golgi apparatus, while encephalomyocarditis virus (EMCV) 2B protein only significantly decreased Ca^2+^ concentration in the ER (12). In contrast, other studies have shown that the expression of hepatitis A virus (HAV) and FMDV 2B proteins increased cytoplasmic Ca^2+^ levels but did not alter the levels of Ca^2+^ stored in organelles (3, 12). Specifically, the experimental results of Xie et al. suggest that the enterovirus (EV) 71 2B protein may mediate Cl^-^ dependent currents in African Xenopus oocytes (14). The reason for this 2B-induced ion concentration variation among different small ribonucleic acid viruses may stem from differences in the proteins themselves and experimental settings and systems, and no studies are showing the permeability of other 2B proteins to Cl^-^. Therefore, further analysis of 2B protein-mediated ion permeability under viral infection and separate expression is required. Also significant for Ca^2+^ homeostasis is the RV NSP4 protein, whose effect on Ca^2+^ levels, along with that of the enterovirus 2B protein, has been characterized using fluorescent Ca^2+^ imaging (233). NSP4 is an ER transmembrane glycoprotein that activates ER calcium sensor matrix interaction molecule 1 (STIM1), leading to the plasma membrane (PM) Ca^2+^ inward flow (190). NSP4 also elevates cellular Ca^2+^ levels through a phospholipase C (PLC)-independent pathway, suggesting that it disrupts Ca^2+^ homeostasis and releases Ca^2+^ channels (189).

Experimental results suggest that viroporin E protein may play an important role in regulating ion homeostasis and the microenvironment of host cells (234, 235). Westerbeck et al. found that IBV E protein transient overexpression alters Golgi pH, providing the first evidence of a coronavirus-mediated alteration of the secretory pathway tubular microenvironment to facilitate infectious virus production (32). A recent study showed that the intracellular expression of SARS-CoV-2 E protein increases intra-Golgi PH (21). IAV M2 also downregulates the expression and function of two host ion channels, the amiloride-sensitive epithelial sodium channel (ENaC) (236) and the cystic fibrosis transmembrane conductance regulator (CFTR) (237) chloride channel, which promotes influenza pathogenesis.

In addition to altering the permeability of the invading cells themselves, cell permeabilization by PV 2B viroporin triggers bystander permeabilization in neighboring cells through a mechanism involving gap junctions, and proteins from MHV E, sindbis virus (SINV) 6K, and HCV NS4A are also able to penetrate neighboring cells to varying degrees (238). Some viroporins (e.g., PV 2B (5)and 3A (103, 104), IBV E (29)) are also able to target intracellular compartments that affect pH or Ca^2+^ homeostasis, thereby blocking protein secretion.

### 3.2 Regulation of the Activation of the Inflammasome

The inflammasome is a molecular platform activated upon cellular infection or stress, triggering the maturation of pro-inflammatory cytokines such as IL-1β, and participating in innate immune defense. The NACHT, LRR, and PYD domain-containing protein 3 (NLRP3) inflammasome is an oligomeric complex composed of the NOD-like receptor NLRP3, the adaptor protein ASC, and Caspase-1 (229). This complex is critical in the host antiviral immune response as it promotes IL-1β and IL-18 secretion and induces pyroptosis (239). Increasing evidence suggests that the effect of viroporins on membrane permeability and the subsequent disruption of ion homeostasis in cellular compartments may be the activation signal required to activate the NLRP3 inflammasome and produce IL-1β and IL-18 (**Figure 3**). This highlights the important role of ion concentration imbalance caused by the ion channel activity of viroporin in activating the NLRP3 inflammasome. Viroporins such as FMDV and HRV 2B protein (93, 114), respiratory syncytial virus (RSV) SH protein (66), IAV M2 protein (140), EMCV 2B protein (109), SARS-CoV E protein (122), SARS 3a protein (126), and HCV p7 protein (168) was reported to activate the NLRP3 inflammasome by disturbing intracellular ion concentrations (K^+^, Ca^2+^, Na^+^). The 2B protein from a variety of picornaviruses (including EMCV, PV, and EV 71), the DNEV NS2A protein and the SARS-Cov E protein were shown to induce NLRP3 cytoplasmic relocalization and inflammasome activation in an intracellular Ca^2+^-mediated manner (58, 109, 122). Other experimental results have shown that influenza virus M2 proton channel activates the NLRP3 inflammasome pathway by regulating intracellular K^+^, Na^+^, and Ca^2+^ concentrations (140).

In addition to disrupting intracellular ion concentrations, viroporins activate the inflammasome in other ways, resulting in an inflammatory response. DNEV NS2A and NS2B protein expression increase apoptosis-associated speck-like protein-containing caspase recruitment domain (ASC) oligomerization and secretion of IL-1β through caspase 1 activation (58). SARS 3a activates NLRP3 inflammasome by inducing disruption of intracellular ion concentration, mitochondrial damage, and TNF receptor-associated factor 3 (TRAF3)-mediated ubiquitination of ASC (126, 128, 131). Paradoxically, Siu et al. found that the oligomerization of ORF 3a was dispensable for the activation of NF-κB or NLRP3 inflammasome, indicating how the viral ion channel protein works in an ion channel-independent manner (128). In contrast, results from another related study showed that SARS-CoV 3a activates the NLRP3 inflammasome in an ion channel-dependent manner (126). This discrepancy may require further studies in the same experimental system to resolve. In addition to this, 3a upregulates the production of pro-inflammatory cytokines and chemokines by activating C-Jun N-terminal kinase (JNK) and the transcription factor nuclear factor-kappa B (NF-kappaB) (129). In contrast, human metapneumovirus (hMPV) SH has been reported to inhibit NF-κB transcriptional activity in airway epithelial cells (184). Other studies have shown that RSV SH viroporin accumulates in the Golgi apparatus within lipid raft structures and may form ion channels that trigger NLRP3 translocation from the cytoplasm to the Golgi apparatus and activate NLRP3 inflammasome (66). HCV-p7 induces the molecular mechanism of SOCS3 through STAT3 and ERK activation and has been shown to inhibit p7 in response to TNF-α pro-inflammatory response (165).

The IAV PB1-F2 protein has a special performance in regulating inflammatory response, exhibiting two distinct roles in promoting and inhibiting NLRP3 inflammasome. On the one hand, PB1-F2 can be integrated into the phagocytic lysosomal compartment triggering NLRP3 inflammasome activation, inducing the secretion of the IL-1β, which leads to severe pathophysiology (153). Several experimental results have shown that PB1-F2 activates NLRP3 inflammasomes and NLRP3-dependent cell recruitment (154), inducing lung inflammation (152). On the other hand, the PB1-F2 protein inhibits the activation of the RLRP3 inflammasome under certain conditions (155). The highly pathogenic H7N9 PB1-F2 protein selectively inhibits RNA-induced NLRP3 inflammasome activation by inhibiting MAVS-NLRP3 interactions in infected cells, but intracellular PB1-F2 does not affect extracellular PB1-F2-induced NLRP3 inflammasome maturation (151). H5N1 and H3N2 PB1-F2 expression reduced IL-1β levels secreted by infected macrophages (150). The intrinsic link between the activation and inhibition of the RLRP3 inflammasome by viroporin PB1-F2 is unclear, but ultimately it is for the better survival and reproduction of the virus in the host cell.

### 3.3 Regulation of Autophagy

Autophagy is a physiological catabolic process in which cells degrade internalized pathogens or worn organelles by forming membrane-enclosed autophagosomes. Although viruses must escape autophagic destruction, some viruses can also disrupt autophagy for their benefit. Studies have shown that viroporins can manipulate autophagy (**Figure 4**) and thus promote viral replication, and among the viroporins that have a strong effect on cellular autophagy are mainly the small ribonucleic acid virus 2B protein, coronavirus 3a protein, influenza virus M2 protein, and rotavirus NSP4 protein. FMDV and CVB3 2B proteins induce robust autophagy in host cells (3, 108). Simultaneous expression of PV 2BC and 3A proteins induces the co-localization of LC3 and LAMP1, which induces autophagy to promote viral replication (106). According to reports, NSP4 can induce autophagy by activating the Ca^2+^/calmodulin-dependent kinase kinase-β (CaMKK-β) signaling pathway or targeting IGF1R (191, 192), and further studies revealed that COPII vesicle transport plays an important role in this (226). Furthermore, early in viral infection, the miRNA encoded by the NSP4 gene targets IGF1R, blocking the PI3K/Akt pathway and triggering autophagy, but ultimately inhibiting autophagic maturation (193).

SARS-CoV 3a proteins can both induce autophagy by inserting into the lysosomal membrane, causing lysosomal damage and dysfunction (Yuan 131), and inhibit autophagosome degradation and thus cellular autophagy by preventing the assembly of the SNARE complex required for HOPS complex-mediated autolysosome formation (132). By comparison, it is found that 3a needs its viroporin activity to promote autophagy, while whether preventing autophagy also needs to be further studied. Similarly, the influenza virus M2 protein also exhibits both autophagy-promoting and autophagy-inhibiting aspects. IAV M2 inhibits autophagy by directly interacting with the autophagy proteins LC3 or Beclin-1, blocking the fusion of autophagosomes and lysosomes (141–143). In contrast, Wang et al. showed that M2 triggers extracellular Ca^2+^ influx-dependent ROS production, which subsequently leads to activation of ATG5 and inhibition of AKT/PKB and MTOR activities *via* the class I phosphatidylinositol 3-kinase (PI3K)-AKT-MTOR signaling pathway, ultimately triggering activation of autophagy (145). The inhibition of autophagy by 3a and M2 proteins may be a strategy employed by viruses during the pre-infection phase to survive in the host cell while releasing daughter viral particles from the cell by promoting autophagy after the maturation of their viral particles. At present, further studies on the regulation of host cell autophagy by viroporins other than the above-mentioned proteins are underway.

### 3.4 Regulation of Apoptosis

Apoptosis is a genetically programmed mechanism used by the host to eliminate damaged or unwanted cells by activating the caspases cascade and can be activated through two major signaling pathways: the extrinsic or death receptor-mediated pathway and the intrinsic or mitochondrial pathway. In addition, excessive Ca^2+^ loading in mitochondria can induce apoptosis by causing the opening of permeability transition pores, permeability swelling of mitochondria, and rupture of the outer mitochondrial membrane, which leads to the release of pro-apoptotic factors such as cytochrome C (230). Some virus-encoded viroporins can promote their propagation by affecting host cell apoptosis (**Figure 5**).

Accumulating evidence suggests that picornavirus 2B proteins can regulate host cell apoptotic responses in multiple ways. HRV16 2B protein activates PERK and ATF6 but not IRE1 to trigger ER stress (15). EV 71 2B protein induces apoptosis by recruiting and directly interacting with the pro-apoptotic protein Bax or by regulating the redistribution and activation of Bax (13, 111). Furthermore, PV 2B protein induces permeabilization of the plasma membrane, triggering apoptosis through the mitochondrial pathway (102). CVB 2B proteins play a major role in inhibiting apoptotic host cell responses by manipulating intracellular Ca^2+^ homeostasis (9). Other viroporins also regulate host cell apoptosis in different ways. Studies have shown that overexpression of the E protein of the family *Coronaviridae* can induce apoptosis (124, 137) and the SARS-CoV E protein can interact with Bcl-xL, making the anti-apoptotic protein Bcl-xL unable to function normally (124). SARS-CoV E activates ER stress and induces pro-inflammatory cytokines to promote apoptosis during late infection (137). In contrast, DeDiego et al. used a microarray-based approach to show that SARS-CoV E may also have anti-apoptotic effects during infection (125). The 3a and 8a proteins of SARS-CoV also induce apoptosis (27, 134). The 8a protein is located in mitochondria, where it can perturb mitochondrial membrane potential and induce apoptosis through a caspase-3-dependent pathway (27).

In addition, the influenza virus M2 protein induces host cell apoptosis by blocking autophagosome maturation (148). It has been reported that Hsp40 acts as a regulator of PKR signaling by interacting with the PKR cytostatic p58^IPK^. Guan et al. found that M2 protein interacts with Hsp40 both *in vitro* and *in vivo*, speculating that it may enhance PKR autophosphorylation by forming a stable complex with Hsp40 and P58^IPK^, thereby inducing cell death (149). Another viroporin PB1-F2 encoded by the influenza virus interacts with two mitochondrial proteins, adenine nucleotide transporter (ANT3) and voltage-dependent anion channel 1 (VDAC1), which are present in the inner and outer mitochondrial membranes, respectively, leading to dissipation of mitochondrial membrane potential, inducing cell death (156). In addition, the PB1-F2 protein can also translocate to the inner mitochondrial membrane space *via* the TOMM40 channel, resulting in a decrease in mitochondrial membrane potential (MMP) and induction of apoptosis (155). In contrast, the BM2 protein interacts with p53 and inhibits its transcriptional and apoptotic activities (164). Some other viroporins (e.g., human respiratory syncytial virus SH protein, HPV E5 protein) have also been reported to have anti-apoptotic activity (174, 176, 182, 200).

### 3.5 Regulation of the Host Cellular Immune Response

The innate immune response is the first line of defense against viral infection, and to break through this line of defense and replicate effectively *in vivo*, many virus-encoded viroporins disrupt host immune defenses in a variety of ways (**Figure 6**). The regulation of host cell immune response by viroporins is mainly manifested as antagonism.

#### 3.5.1 Identification of Interfering PRRs

Recognition of pathogens is mainly mediated by pattern recognition receptors (PRRs), including Toll-like receptors (TLRs), nucleotide oligomerization domain-like receptors (NLRs), cyclic GMP-AMP synthase (cGAS), and retinoic acid-inducible gene-1-like receptors (RLRs), which recognize pathogenic microbial infections and form the corresponding signal transduction to generate an immune response. RIG-I-like receptors (RLRs), including RIG-I, melanoma differentiation-associated protein (MDA)-5, and LGP2, are a series of cytoplasmic RNA unwinds that detect multiple viral RNAs accumulated during viral infection or replication. Activation of RIG-I is responsible for the induction of type I interferons (IFNs) and the expression of many cytokines and chemokines (240). To survive and multiply in host cells, viroporins can interfere with their viral recognition by PRRs.

It was shown that FMDV 2B protein interacts with and inhibits the expression of RIG-I and MDA5 to antagonize their mediated antiviral effects (97). In 2019, Liu H et al. found that FMDV infection triggered NOD2 transcription and reduced NOD2 protein expression. Further experimental results showed that 2B protein was one of the reasons for this phenomenon (95). Similarly, the IAV PB1-F2 protein exhibited type I IFN antagonism by interfering with the RIG-I/MAVS complex (160). The work of Lateef et al. showed that Toll-like receptors were targets of norovirus (NV) NS1/2, which decreased the expression of TLR-4, -7, -8, and -9 and increased the expression of several pro-inflammatory cytokines/chemokines (186). To avoid detection of mitochondrial DNA during infection, the dengue virus NS2B protein targets the DNA sensor cyclic GMP-AMP synthase (cGAS) for lysosomal degradation and results in the inhibition of type I interferon production in infected cells (172). Whether other viroporins are also involved in regulating host cell immune responses by interfering with PRRs recognition is currently under further investigation.

#### 3.5.2 Interference With Adaptor Molecules, Kinases, and Downstream Effectors in Innate Immune Signaling Pathways

Virus-encoded viroporins have developed multiple mechanisms to disrupt the recruitment process or degrade linker molecules, as well as related kinases critical for signal transduction, to block subsequent signal transduction and antagonize the host antiviral response. It has been reported that phosphorylation of RIP2 was identified as a marker for activation of the NOD2-mediated NF-κB pathway (241). In 2021, Liu H et al. found that FMDV infection triggered the transcription of RIP2, and its 2B protein could reduce the expression of RIP2 protein (95). Both FMDV 2B and DENV NS2A inhibit the phosphorylation of TBK1 and IRF3 in the RLR signaling pathway (96, 170). Furthermore, DENV NS2A and NS2B proteins inhibit the production of type I IFN by cleaving STING (171, 173). According to literature reports, influenza virus-encoded viroporin PB1-F2 is a particularly potent inhibitor of antiviral signaling, inhibiting the expression of mitochondrial antiviral signaling protein (MAVS) and it is signaling through various means. PB1-F2 can degrade MAVS by inducing mitophagy and lead to the inhibition of type I interferon production (162, 163). Cheung et al. found that PB1-F2 protein interacts with TRIM31-MAVS by forming protein aggregates on mitochondria, thereby preventing K63-polyubiquitination and MAVS aggregation and promoting MAVS degradation, ultimately inhibiting host antiviral defense (161). In addition to this, an increasing number of studies have shown that the reduction of mitochondrial inner membrane potential is also an important way in which PB1-F2 protein inhibits MAVS signaling (155, 159). Several other studies have reported that HIV-1 Vpu effectively inhibits NF-κB-induced antiviral immune responses at the transcriptional level (180). HAV 2B is able to interfere with IRF-3 phosphorylation and inhibit IFNβ gene transcription (115).

In addition, some other viroporins (e.g., BTV NS3, RSV SH) resist the host defense response by interacting with BRAF or inhibiting p65 phosphorylation (183, 194). In particular, the influenza virus M2 protein antagonizes autophagy and interacts with MAVS, thereby increasing MAVS aggregation, positively regulating MAVS-mediated antiviral innate immune responses (145), however, overactivation of MAVS signaling could lead to detrimental levels of inflammation or other immunopathological consequences that are harmful to the host.

#### 3.5.3 Interference With IFN-Mediated Signaling

Interferons play a crucial role in regulating and activating the host innate immune response to viral infection and limiting viral replication and spread and are also important targets for viroporins. Experimental results have shown that HMPV SH proteins downregulate type I IFN pathway signaling by affecting STAT1 expression and phosphorylation (185). Similarly, DENV NS2A, NS4A, and NS4B proteins complex together to block STAT1 phosphorylation and inhibit ISG production (169). BTV NS3 and NS4 synergistically antagonize type I interferon signaling by targeting STAT1 (195). In addition, BTV NS3 blocks IFN signaling by binding STAT2 to induce its degradation through an autophagy-dependent mechanism (196). Karyopherins (KPNAs) are cytoplasmic proteins essential for nuclear transport of p-STAT1/2 and translocation of ISGF complexes, and enterovirus A71 2B inhibits interferon-activated JAK/STAT signaling by inducing Caspase-3-dependent KPNA1 degradation (112). Whether other viroporins can also antagonize the host antiviral response by blocking the JAK/STAT pathway is unknown and requires further experimental investigation.

#### 3.5.4 Other Ways to Resist the Host Immune Response

Major histocompatibility complex (MHC) class 1 is also an important player in the antiviral response. Studies have shown that PV 3A proteins can impair MHC class I antigen presentation (105). CVB3, which encodes 2B and 2BC proteins in the same family as PV, upregulates the endocytosis of MHC class I by focusing endocytic vesicles on the Golgi complex and rapidly removes proteins from the cell surface. This may render CD8^+^ T cells unrecognizable to CVB3-infected cells and therefore inaccessible to many antiviral effector molecules, which is important for immune evasion by CVB3 (242). Similarly, PB1-F2 induces IFNβ through the NF-κB pathway independently of the AP-1 and IRF3 pathways, and overexpression of the MHC-I gene is involved in suppressing antiviral immunity (157, 158). The interaction between NV NS1/2 and the vesicle-associated membrane protein VAP-A suggests that calicivirus manipulate intracellular trafficking, thereby inhibiting or blocking key innate immune proteins (TLR, IFN, MHC, etc.) to the cell surface (187, 188).

Vpu downregulates bone stromal cell antigen 2 (BST-2) protein from the cell surface, which protects HIV-infected CD4^+^ T cells from antibody-mediated cell lysis in addition to promoting self-release (63, 64), it also protects HIV-infected CD4^+^ T cells from antibody-mediated cell lysis (177). It should be noted that mutants that impair Vpu ion channels do not affect the downregulation of BST-2, suggesting that the downregulation of BST-2 is independent of the ion channel activity of Vpu (243). In addition, Vpu promotes phagocytosis of infected CD4^+^ T cells by macrophages through downregulation of CD47 to facilitate viral dissemination and promote immune evasion (62).

Cytoplasmic mitochondrial DNA (mtDNA) activates cGAS-mediated antiviral immune responses, and the viroporin activity of influenza virus M2 or EMCV 2B proteins triggers mtDNA translocation into the cytoplasm in a MAVS-dependent manner. Although influenza virus-induced cytoplasmic mtDNA stimulates cGAS and DDX41-dependent innate immune responses, influenza virus nonstructural protein 1 (NS1) binds to mtDNA to evade STING-dependent antiviral immunity (110). A recent study demonstrated that Cyclophilin A (CypA) plays a role in promoting RIG-I-mediated antiviral immune responses by controlling the ubiquitination of RIG-I and mitochondrial antiviral signaling protein (MAVS) (244). FMDV 2B attenuates CypA during FMDV infection by interacting with CypA mediated antiviral effects during FMDV infection, and further studies have shown that this interaction is specific to FMDV 2B in picornavirus viroporins (94). In addition to this, some other viroporins evade the host antiviral response by some other means (**Table 2**).

## 4 Viroporins Interact With Other Host or Viral Proteins to Promote Their Own Proliferation

Viruses lack the necessary machinery for self-replication and therefore rely on host cell machinery for reproduction. Many viruses utilize the host cell replication machinery to establish infection through host-virus protein-protein interactions (PPIs) (245). Some viruses encode viroporins that interact with host proteins to ensure the successful completion of their life cycle (**Table 3**).

**Table 3:** The role of viroporins interacting with other host or viral proteins in the viral life cycle.

The role of viroporin interacting with host protein/viral protein in the viral life cycle	Virus or host protein	Virus-viroporin	Regions of viroporin required for interaction	Is it related to viroporin activity	References
Virus uncoating	Transportin-3	IAV-M2	–	–	(246)
Viral replication	EEF1G	FMDV -2B	–	–	(247)
	GPS1	IAV-M2	–	–	(248)
	ACBD3	PV-3A	C-terminus of the cytoplasmic domain	–	(249, 250)
	PV-2C	PV-2B	–	–	(251)
		PV-3A	–	–	(251)
	Microtubules	RV-NSP4	C-terminal 129 -175aa	NO	(252)
	Caveolin-1	IAV-M2	Cytoplasmic domain	NO	(253)
	PB1	IAV-PB1-F2	–	–	(254)
	HAX-1	IAV-PB1-F2	C-terminal 1 - 50 aa	Yes	(255)
	VAPA	NV- NS1/2	FFAT motif	NO	(188)
	JCV-T antigen	JCV -gnoprotein	N-terminal	Yes	(256)
Viral replication, Viral release	SARS-CoV -nsp3	SARS-CoV E	–	–	(257)
Viral assembly	TRAPPC6A, TRAPPC6AΔ	IAV- M2	Leucine residue at position 96	NO	(258)
	Caveolin-1	RV-NSP4	114-135aa	–	(259)
	Cyclin D3	IAV-M2	CTD	–	(260)
	BAP31	HPV-E5	C-terminal	NO	(261)
	IBV -M	IBV E	37-57aa	NO	(262)
	SARS-CoV-S	SARS-CoV-E	–	–	(263)
Viral assembly, Viral release	Caveolin-1	SARS-CoV-3a	Cytoplasmic domain	NO	(264)
	Cyclin D3	IAV-M2	Cytoplasmic domain	NO	(260)
Viral release	Tetherin	HIV-Vpu	CTD	Yes	(265)
	LIS1	PV-3A	–	–	(266)
	UBR4	IAV-M2	TMD and C-terminal	Yes	(267)
	AnxA6	IAV-M2	CTD	NO	(268)
	Tetherin	IAV-M2	Extracellular and transmembrane structural domains	Yes	(269)
	MARCH 8	IAV-M2	K63	NO	(270)
	Tsg101	BTV-NS3	PSAP motif	NO	(271)
	S100A10/p11	BTV-NS3	N-terminal 13 residues	NO	(272)
	Viperin	RV-NSP4	C-terminal	–	(273)
	Heterochromatin Protein-1α(HP-1α)	JCPyV -gnoprotein	N-terminal 24 aa	–	(274)
	FEZ1	JCPyV -gnoprotein	–	–	(275)
	PARP	SV40-VP3	N-terminal 35 aa	–	(276)
Not Determined	PALS1	SARS-CoV E	C-terminal	NO	(277)
	SARS-CoV-7a	SARS-CoV E	–	–	(278)
	ATP1A1 and Stomatin	SARS-CoV E	–	–	(22)
	ATP1B1	IAV-M2	Cytoplasmic domain 28-48aa	NO	(46)
		IBV-M2		NO	
	BAP31	RSV-SH	N-terminal α-helix	–	(279)
	Importin β1, Importin 7	BEFV-α1	C-terminal	NO	(77)

“-” represents “unidentified.” CTD, Cytoplasmic tail domain.

Current research data suggest that viroporins interact with host proteins and are mainly involved in regulating the later stages of the viral life cycle. Relevant studies have shown that viroporins such as Picornavirus 2B and 3A proteins, IAV M2 and PB1-F2 proteins, and coronavirus E protein can regulate the replication stage of the virus by interacting with different host proteins, of which influenza virus AM2 and PB1-F2 proteins are good examples. The M2 protein has been reported to interact with the G protein pathway repressor protein 1 (GPS1), which is involved in the transcription and replication of influenza virus genomic RNA by activating the NF-κB signaling pathway (248). Mazur et al. found that the PB1-F2 protein co-localizes and directly interacts with the viral PB1 polymerase protein, thereby determining the localization of the PB1 protein and enhancing viral polymerase activity, which ultimately leads to enhanced accumulation of viral RNA (254). Recent experimental results identified HCLS1-associated protein X1 (HAX-1) as a functional restriction factor of IAV polymerase that binds to PA subunits, and PB1-F2 protein promotes its efficient replication by binding to HAX-1 and relieving HAX-1-mediated restriction of avian viral polymerase PA (255). In addition, FMDV 2B and PV 3A proteins interact with eukaryotic translation elongation factor 1γ (eEF1G) and acyl-CoA-binding domain protein 3 (ACBD3), respectively, to promote self-replication. Further studies revealed that eEF1G may play a potential role in assisting protein 2B to produce virus-induced vesicles and induce cell lysis (247). ACBD3 is a Golgi-resident protein involved in maintaining Golgi structure and regulating intracellular trafficking between the ER and the Golgi (280). PV 3A interacts with it and counteracts the inhibitory effect of ACBD3 on viral replication (250). Several other studies have shown that 3A proteins can utilize ACBD3 to recruit PI4KIIIβ to viral replication sites to facilitate self-replication (249, 281). Furthermore, NV NS1/2 interacts with the host protein VAMP-associated protein A (VAPA) to promote self-replication (188).

After virus replication is completed and enters the assembly stage, viroporins such as influenza virus M2 protein and RV NSP4 protein play an important regulatory role by binding to host factors. Cyclin D3 is a key cell cycle regulator in the G0/G1 phase, and the M2 protein binds to it, inducing its redistribution from the nucleus to the cytoplasm and subsequent degradation by the proteasome, a process that facilitates the correct assembly of progeny virions (260). Caveolae are known to serve as a site for signal transduction, viral entry into cells, and viral assembly. Experimental results found that NSP4 interacts directly with caveolin-1 to facilitate the intracellular transport of NSP4 from the ER to the cell surface (259), and Sapin et al. suggested that this interaction may contribute to the final step in RV morphogenesis (282). Similarly, the coronavirus 3a protein plays an important role in assembly and release by interacting with the caveolin-1 protein (264). In addition, the HPV E5 protein can interact with BAP31 and can mediate post-transcriptional effects or promote viral particle assembly (261).

After the virus matures, its encoded viroporins can release the mature particles extracellularly by binding to host proteins. Currently, influenza virus M2 protein, BTV NS3 protein and JCV gnoprotein protein are more studied in this regard. It has been found that the influenza virus M2 protein binds to the ubiquitin-protein ligase E3 component N-recognition protein 4 (UBR4) or Tetherin (BST-2) protein and facilitates the release of the virus outside the cell by promoting apical translocation of viral proteins or by downregulating Tetherin through the proteasomal pathway (267, 269). BTV NS3 assists in the viral release by recruiting the ESCRT-I protein Tsg101 or interacting with the S100A10/p11 protein (271, 272). JCV gnoprotein promotes the release of progeny viruses by interacting with heterochromatin protein-1α (HP-1α) or fiber bundle and elongation protein zeta1 (FEZ1) (275). Furthermore, studies have reported that Vpu counteracts the viral release-limiting effect of tetherin by interacting with tetherin and downregulating tetherin in the plasma membrane (265). In contrast, some studies have found that host proteins inhibit viral release by binding to viroporins. The human protein annexin A6 (AnxA6) interacts with M2 and negatively regulates influenza virus infection by affecting viral budding (268). The membrane-associated RING-CH8 (MARCH 8) protein inhibits IAV release by redirecting the viral M2 protein from the plasma membrane to the lysosome for degradation (270). Similarly, the IFN-stimulating protein Viperin delays rotavirus release by inhibiting NSP4-induced intrinsic apoptosis (273). This negative regulation of viral release mediated by host proteins through interaction with viroporins may be a strategy for the host to combat viral infection, but the detailed molecular mechanisms need to be further investigated.

In addition to interacting with host proteins, viroporins have also been reported to bind to their other viral proteins to promote the completion of the viral life cycle. For example, both mammalian and yeast two-hybrid systems have shown that PV 3A multimerizes and interacts with 2B and 2C ATPases to promote viral replication (251). IBV E and M proteins interact through their cytoplasmic tails leading to the assembly of coronavirus-like particles (262). Recent studies have shown that the SARS-CoV E protein induces the retention of the S protein in the ERGIC by regulating the cellular secretory pathway, preventing the formation of syncytia, and promoting the assembly of SARS-CoV viral particles (263). The current growing family of viroporin and consequently the increasing number of host proteins interacting with them have been reported, and such interactions play an important role in regulating the viral life cycle and promoting viral proliferation, but the specific stages of the viral life cycle at which they act, and the mechanisms remain to be further investigated.

## 5 Conclusions

Viroporins are potential targets for antiviral therapy because of their significant impact on the viral life cycle and host and have become targets for inhibitory drug development and antiviral therapy. AM2, the first viroporin discovered, has a well-established biological role in viral pathogenesis and is a proven drug target (283). Amantadine is the first drug to inhibit influenza virus replication by inhibiting the ion channel activity of M2, which can mediate the conversion of hemagglutinin (HA) to its low pH conformation by interacting with the AM2 protein, thereby preventing proton conduction and inhibits viral entry (284). Amantadine has also been reported to have a targeted inhibitory effect on viroporins such as FMDV 2B, SARS-CoV E, HCV p7 (3, 54, 121), however, drug resistance limits its clinical application. The identification and application of tauroursodeoxycholic acid (TUDCA) as an M2 proton channel inhibitor will expand our understanding of IAV biology and complement the current anti-IAV arsenal (285). Subsequent experimental results showed that drugs such as hexamethylene amiloride (HMA) (286), gliclazide (120), memantine (120), and emodin (127) have inhibitory effects on the channel activity of some viroporins (**Table 2**). Recent studies have demonstrated synergistic antiviral effects achieved by the combination of adamantanes and novel compounds (287), which provides directions for the development of viroporins inhibitory drugs. Currently, some other pharmacological inhibitors against viroporins (e.g., pyrin B, BIT225, DIDS, and divalent copper complexes) have been reported (14, 166, 181, 288). However, none of these drugs has an effective inhibitory effect against most viroporin activity at this stage, and therefore, new anti-viroporins drugs are urgently needed to be developed. In addition to antiviral drug research, attenuated viruses lacking viroporins are increasingly being suggested as vaccine candidates (289–291).

Although viroporins are increasingly studied, their protein families are expanding, and their functions are better understood, many aspects remain unclear, and future studies on this area should continue to elucidate the structure, function, and mechanism of such viral proteins to develop a library of antiviral targets across multiple virus families.

## Author Contributions

XX and AC contributed to the design of the manuscript. XO, DS, SM, JH, QY, YW, SC, SZ, and DZ, provided ideas contributing to the conception of this manuscript. RJ, ML, X-XZ, QG, and BT helped to create the figures. MW modified the manuscript. All the authors reviewed the manuscript. All authors contributed to the article and approved the submitted version.

## Funding

This work was supported by the China Agriculture Research System of MOF and MARA, and the Sichuan Veterinary Medicine and Drug Innovation Group of China Agricultural Research System (SCCXTD-2020-18).

## Conflict of Interest

The authors declare that the research was conducted in the absence of any commercial or financial relationships that could be construed as a potential conflict of interest.

## Publisher’s Note

All claims expressed in this article are solely those of the authors and do not necessarily represent those of their affiliated organizations, or those of the publisher, the editors and the reviewers. Any product that may be evaluated in this article, or claim that may be made by its manufacturer, is not guaranteed or endorsed by the publisher.
